# Blood pressure changes during 22-year of follow-up in large general population - the HUNT Study, Norway

**DOI:** 10.1186/s12872-016-0257-8

**Published:** 2016-05-12

**Authors:** Jostein Holmen, Turid Lingaas Holmen, Aage Tverdal, Oddgeir Lingaas Holmen, Erik R. Sund, Kristian Midthjell

**Affiliations:** HUNT Research Centre, Department of Public Health and General Practice, Norwegian University of Science and Technology (NTNU), Forskningsvegen 2, 7600 Levanger, Norway; Division of Epidemiology, Norwegian Institute of Public Health, Oslo, Norway

## Abstract

**Background:**

While hypertension still is a major health problem worldwide, some studies have indicated that the blood pressure level has decreased in some populations. This population based cohort study aims at analysing blood pressure changes in a large Norwegian population over a 22 year period.

**Methods:**

Data is acquired from three comprehensive health surveys of the HUNT Study conducted from 1984–86 to 2006–08. All citizens of Nord-Trøndelag County, Norway, >20 years were invited: 74,549 individuals participated in 1984–86; 64,523 in 1995–97; and 43,905 in 2006–08.

**Results:**

Both systolic and diastolic blood pressure levels decreased substantially from mid 1980s to mid 2000s, with the most pronounced decrease from 1995–97 to 2006–08 (from 136.0/78.9 to 128.3/70.9 mmHg in women and from 140.1/82.1 to 133.7/76.5 mmHg in men). Although the use of blood pressure lowering medication increased, there was a considerable decrease even in those who reported never use of medication (mean decrease 6.8/7.2 mmHg in women and 6.3/5.3 mmHg in men), and the decrease was most pronounced in the elderly (mean decrease 16.1/12.4 mmHg in women and 14.7/10.4 mmHg in men aged 80+). Mean heart rate, total cholesterol and daily smoking decreased, self-reported hard physical activity increased, while body weight and the prevalence of diabetes increased during the same period.

**Conclusions:**

The BP decrease might seem paradoxically, as body weight and prevalence of diabetes increased during the same period. Salt consumption might have decreased, but no salt data is available. The parallel decrease in mean heart rate might indicate reduction in the white-coat phenomenon, or increased use of beta blockers or calcium channel blockers for other diagnosis than hypertension. Additionally, the data could support the “healthy obese” hypothesis, i.e., that subgroups in the population can sustain obesity without serious health consequences.

**Electronic supplementary material:**

The online version of this article (doi:10.1186/s12872-016-0257-8) contains supplementary material, which is available to authorized users.

## Background

A large number of studies have shown the health benefits of lowering of blood pressure (BP) and the efficacy of specific lifestyle and pharmacological interventions, but hypertension is still a major health problem world-wide. In a study covering 5.4 million participants, mean systolic blood pressure (SBP) decreased by only 1 mmHg per decade for men and women between 1980 and 2008, and in some countries SBP had increased [1]. Western Europe and Australasia showed a more favorable trend; SBP decreased by 3.5 mmHg or more per decade in women and more than 2.0 mmHg per decade in men. In the WHO MONICA study, following 38 European populations from mid 1980s to mid 1990s, SBP decreased 2.2 mmHg in men and 3.3 mmHg in women, and diastolic blood pressure (DBP) decreased 1.4 mmHg in men and 2.2 mmHg in women [2]. However, despite that antihypertensive medication increased, no effect from improving treatment of hypertension was detected, and other determinants of the BP decrease was therefore suggested to have had impact in the populations. Except the well-known effect of drug treatment, little has been achieved in understanding the determinants of BP trends on a population level [3].

The aim of the present study was therefore to improve the understanding of the population BP changes. We systematically assessed both SBP and DBP changes in a large population over a 22 year period. Having collected a number of health- and other background data, we have analyzed the nature of the BP changes in all age groups 20 years and over, special emphasizing on people who have never taken BP medication.

## Methods

We included individuals from the Nord-Trøndelag Health Study (HUNT), a population-based health study with medical histories on approximately 120,000 individuals from Nord-Trøndelag County, Norway, collected in three surveys (HUNT1, 2, and 3) [4–6]. All residents of the county aged 20 years and older were invited to participate. Self-reported questionnaires, clinical examination and non-fasting venous blood samples (HUNT2 and HUNT3) were collected in 1984–86 (HUNT1, *N* = 74,549, 88 % of invited), 1995–97 (HUNT2, *N* = 64,523, 71 % of invited) and 2006–08 (HUNT3, *N* = 43,905, 59 % of invited) (Table 1). Totally, 27,605 individuals participated in all three surveys. The population of Nord-Trøndelag County (*n* ~ 130,000) is stable and homogenous, and is representative of the Norwegian population regarding demography, socioeconomic factors, morbidity and mortality [5]. After the HUNT3 survey a comprehensive non-participation study was conducted, analysing the impact of the decreasing participation rate [7].

**Table 1:** Clinical characteristics of participants in the three surveys of The HUNT Study, Norway: HUNT1 (1984–86), HUNT2 (1995–997) and HUNT3 (2006–08)

	HUNT1	HUNT2	HUNT3
	(1984–86)	(1995–97)	(2006–08)
Participants (n)	74,549	64,523	43,905
Age, years (mean, SD)	49.1 (18.0)	50.4 (17.4)	53.1 (16.1)
Female (%)	50.7	53.2	54.6
Systolic BP, mmHg (mean, SD)	136.8 (23.3)	137.9 (21.8)	130.7 (18.6)
Diastolic BP, mmHg (mean, SD)	84.0 (11.7)	80.3 (12.2)	73.4 (11.2)
BP medication now/previosly (%)	13.0	13.7	20.9
BMI, kg/m^2^ (mean, SD)	25.2 (3.9)	26.4 (3.9)	27.2 (4.4)
Heart rate, beats/min (mean, SD)	74.9 (12.6)	73.2 (12.7)	70.2 (11.5)
Myocardial infarction (%)	2.6	3.3	3.2
Stroke (%)	1.8	1.9	2.6
Diabetes (%)	2.9	3.1	4.4
Se-Total chol, mmol/L (mean, SD)		5.89 (1.26)	5.49 (1.11)
Se-HDL chol, mmol/L (mean, SD)		1.38 (0.39)	1.35 (0.35)
Se-Triglycerides,mmol/L (mean, SD)		1.76 (1.14)	1.63 (1.01)
Se-Creatinine, mmol/L (mean, SD)		88.03 (15.77)	81.82 (18.76)
Se-Glucose, mmol/L (mean, SD)		5.47 (1.54)	5.59 (1.57)
Waist circumference, cm (mean, SD)		86.5 (11.8)	93.6 (12.3)

### Questionnaires

Each participant completed two extensive questionnaires. First, they received a questionnaire by mail prior to attending the clinical exam regarding health and life style factors, including blood pressure treatment, information of cardiovascular related diseases and diabetes, physical exercise and smoking. At attendance, they were handed a second questionnaire to be completed at home and returned by mail mainly regarding self-reported diseases [6]. For this study, we use the following questions (answering alternatives in parenthesis): *Are you taking medication for high blood pressure?* (Currently taking medication/Previously, but not now/Have never taken it). *Have you had or do you have: Myocardial infarction (heart attack)?* (Yes/No), *Diabetes?* (Yes/No). *Do you smoke?*(Yes/No). *Daily cigarette smoker?* (Yes/No). *How much of your leisure time have you been physically active during the last year?* (Hours per week: None/Less than 1/1-2/3 or more). *Vigorous physical activity (sweating/out of breath)?* (Yes/No). *What is your highest level of education?* (Primary school 7–10 years, continuation school, folk high school/High school, intermediate school, vocational school, 1–2 years high school/University qualifying examination, junior college, A levels/University or other post-secondary education, less than 4 years/University/College, 4 years or more).

### Measurements

In HUNT1 [4], SBP and DBP was measured manually by specially trained nurses using calibrated mercury manometers. The BP was measured on the right arm with cuffs adjusted according to the arm circumference, and after the participant had been sitting relaxed for five minutes. SBP was recorded at the first Korotkoff sound and the DBP was recorded when the fifth Korotkoff sound disappeared (last sound). SBP and DBP were read twice with one minute interval. Heart rate was counted manually for 15 s and multiplied by four. In HUNT2 [5], the examination procedures were similar as in HUNT1, except that automated measures based on oscillometry was used (Critikon Dinamap 845XT and XL9301, acquired by GE Medical Systems Information Technologies in 2000). SBP and DBP and heart rate were read three times with a one minute interval. In HUNT3 [6], the same protocol and procedures as in HUNT2 were used, including Dinamap devices (XL9301 and Critikon 8100). For practical purposes only two BP and heart rate readings were performed in a sample of HUNT3 participants (*n* = 6,188), and they were excluded from the analyses [6]. BP measured with the Dinamap devices used in HUNT2 and 3 are slightly lower than those measured with a sphygmomanometer, especially for DBP [8]. Height and weight were measured using standardized instruments with the participants wearing light clothes without shoes; height to the nearest 1.0 cm and weight to the nearest 0.5 kg. Waist circumference was measured horizontally at the height of the umbilicus to the nearest 1.0 cm.

### Laboratory measurements

For HUNT2 and HUNT3, non-fasting blood samples were drawn whenever the participants attended the screening site. In HUNT2 total serum cholesterol was measured using an enzymatic colorimetric cholinesterase method, HDL by enzymatic colorimetric cholesterol esterase method after precipitation with phosphor tungsten and magnesium ions, non-fasting triglycerides by enzymatic hexokinase method, non-fasting serum glucose by an enzymatic hexokinase method and serum creatinin by Jaffè method [9].

### Statistics

All HUNT data are linked to the unique 11-digit personal identification number given to each Norwegian citizen at birth, enabling linkage of data for each individual. We used the second BP reading in HUNT1, and the mean of the second and third reading in HUNT2 and HUNT3. Body Mass Index (BMI) was calculated according to WHO (kg/m^2^) [10]. Quartiles of total cholesterol (Q1-4) were calculated for HUNT2 (<4.8 mmol/L, 4.8–5.59 mmol/L, 5.6–6.39 mmol/L, and ≥ 6.4 mmol/L, respectively). Quartiles of heart rate (Q1-4) were calculated for HUNT2 (beats/min): Q1: <64.0, Q2: 64.5–71.9, Q3: 72.0–80.9, Q4: ≥ 81.0. Pack years were calculated as years of daily smoking times number of packs smoked per day. Change in BP from HUNT2 to HUNT3 was calculated as delta BP, i.e., BP (mmHg) in HUNT2 minus BP (mmHg) in HUNT3. Delta BMI: BMI (kg/m^2^) in HUNT2 minus BMI (kg/m^2^) at HUNT3, delta Se-cholesterol: total cholesterol (mmol/L) at HUNT2 minus total cholesterol (mmol/L) at HUNT3, delta heart rate (HR): HR (beats/min) at HUNT2 minus HR (beats/min) at HUNT3. We performed linear regression analyses in women and men separately with delta DBP as the dependant variables. *P* < 0.05 was considered significant. Nonparametric kernel-density plots were constructed in Stata (version 13) with the kdensity command and the default Epanechnikov kernel function was utilized. A kernel-density plot is a smoothed representation of a histogram where the area under the curve shows the proportion of values compared to all values and sums to 1. The other statistical analyses were performed using the Statistical Package for the Social Science, version 21.0 (IBM SPSS, New York).

## Results

Valid BP measurements were available on 74,519 individuals from the mid 1980s (HUNT1), 64,523 individuals from the mid 1990s (HUNT2) and 43,905 individuals from mid 2000s (HUNT3). Clinical characteristics of the study participants are summarized in Table 1.

### Blood pressure change in the population

**Fig. 1:**
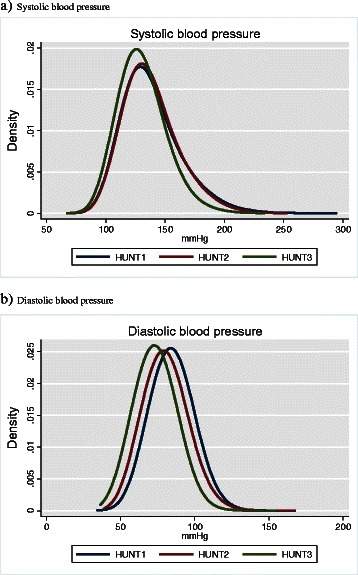
Nonparametric kernel-density estimates for the distribution of systolic (**a**) and diastolic (**b**) blood pressure in HUNT1 (*n* = 74,519), HUNT2 (*n* = 64,523) and HUNT3 (*n* = 43,905)

**Fig. 2:**
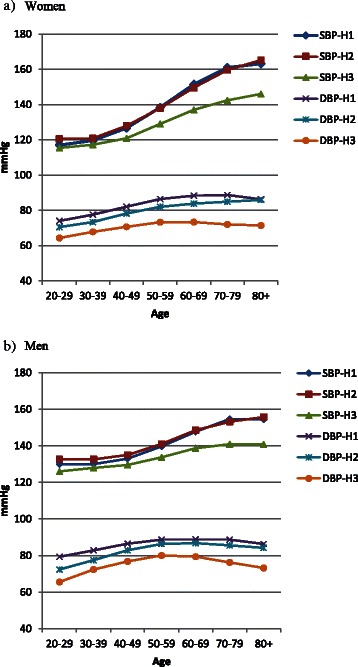
All participants. Mean systolic (SBP) and diastolic (DBP) blood pressure in HUNT1 (H1, 38,173 women, 36,376 men), HUNT2 (H2, 34,325 women, 30,198 men) and HUNT3 (H3, 23,968 women, 19,937 men) in women (**a**) and men (**b**) by age. (Additional file 1: Table S1)

We did not observe change in mean SBP from HUNT1 to HUNT2, while for mean DBP we observed a decrease of 3.7 mmHg for women and 3.4 mmHg for men (from 82.6 mmHg to 78.9 mmHg and from 85.5 mmHg to 82.1 mmHg, respectively). From HUNT2 to HUNT3 mean SBP decreased by 7.7 mmHg in women and 6.4 mmHg in men (from 136.0 mmHg to 128.3 mmHg and from 140.1 mmHg to 133.7 mmHg, respectively) and mean DBP decreased by 8.0 mmHg in women and 5.6 mmHg in men (78.9 mmHg to 70.9 mmHg and 82.1 mmHg to 76.5 mmHg, respectively). Figure 1 illustrates that the distributions of SBP in HUNT1 and HUNT2 were closely overlapping, while SBP in HUNT3 was skewed to the left with the right hand side of the bell curve somewhat compressed. The distribution of DBP showed a consistent and parallel shift to the left for HUNT2 and HUNT3 compared to HUNT1. Stratifying by age illustrates that both the reduction of SBP and DBP mean values increased by age in both women and men, with a maximum decrease in those above 80 years of age (Fig. 2, Additional file 1: Table S1).

**Fig. 3:**
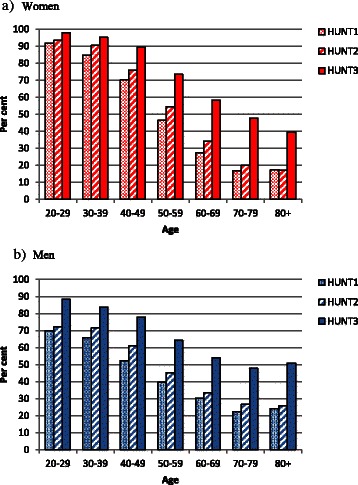
Per cent of participants with SBP <140 mmHg and DBP <90 mmHg in HUNT1, HUNT2 and HUNT3 by age in women (**a**) and in men (**b**)

The distribution of BP according to the ESH/ESC definitions [11] shows a general shift towards the “optimal” and “normal” categories (<120/80 mmHg and 130-139/85-89 mmHg, respectively) for both women and men and in nearly all age groups (Additional file 1: Table S2). The proportion of participants with “normal” BP according to the ESH and ESC Guidelines (SBP below 140 mmHg and DBP below 90 mmHg) [11] increased from 52.8 % in HUNT1 via 57.4 % in HUNT2 to 71.0 % in HUNT3. The improved control increased in both sexes and in all age groups (Fig. 3 and Additional file 1: Table S3).

### Blood pressure lowering medication

**Fig. 4:**
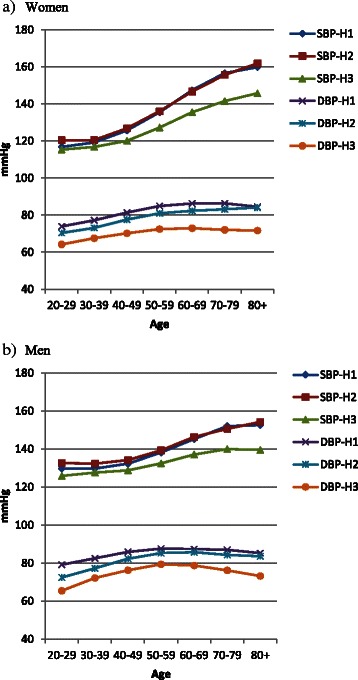
Participants reporting never taking blood pressure medication. Mean systolic (SBP) and diastolic (DBP) in HUNT1 (H1, 32,114 women, 33,000 men), HUNT2 (H2, 29,178 women, 26,350 men) and HUNT3 (H3, 19,106 women, 15,692 men) by age in women (**a**) and in men (**b**). (Additional file 1: Table S4)

The prevalence of self-reported BP lowering drug treatment increased from 12.9 % in HUNT1 via 13.7 % in HUNT2 to 20.6 % in HUNT3, and we observed a decrease in BP for participants who were currently or had previously been under treatment between the three measurements. In accordance with the great increase in self-reported drug treatment from HUNT2 to HUNT3, the decrease was more pronounced in this period (16.2 mmHg for SBP and 11.9 mmHg for DBP) compared to the change observed from HUNT1 to HUNT2 (1.5 mmHg for SBP and 4.8 mmHg for DBP) (Table 2). More surprisingly, we observed a similar (although smaller) trend for those who had never used BP lowering medication: a decrease of 6.7 mmHg in mean SBP and 6.4 mmHg in mean DBP between HUNT2 and HUNT3 (Table 2). For these individuals, the decrease observed in BP between HUNT2 and HUNT3 was more pronounced among the older age groups, (Fig. 4 and Additional file 1: Table S4).

**Table 2:** Mean systolic (SBP) and diastolic (DBP) blood pressure in individuals taking blood pressure medication “now or previously” and in individuals “never” taking blood pressure medication

	Blood pressure medication	n^1^	SBP mmHg	SD	Delta SBP mmHg	DBP mmHg	SD	Delta DBP mmHg
HUNT1	«Now or previously»	9,683	158.0	25.1		93.2	11.3	
	«Never»	65,118	133.7	21.3		82.6	11.2	
HUNT2	«Now or previously»	8,860	156.5	23.3	1.5^a^	88.4	13.1	4.8^a^
	«Never»	55,739	134.9	20.0	−1.2^a^	79.0	11.6	3.6^a^
HUNT3	«Now or previously»	9,089	140.3	19.4	16.2^b^	76.5	11.7	11.9^b^
	«Never»	34,982	128.2	17.5	6.7^b^	72.6	10.9	6.4^b^

Differences in blood pressure (Delta SBP and Delta DBP) between HUNT1 and HUNT2 (^a^) and between HUNT 2 and HUNT 3 (^b^) by blood pressure medication

^1^ Number of participants with valid measures varies slightly

### Individual trends

**Fig. 5:**
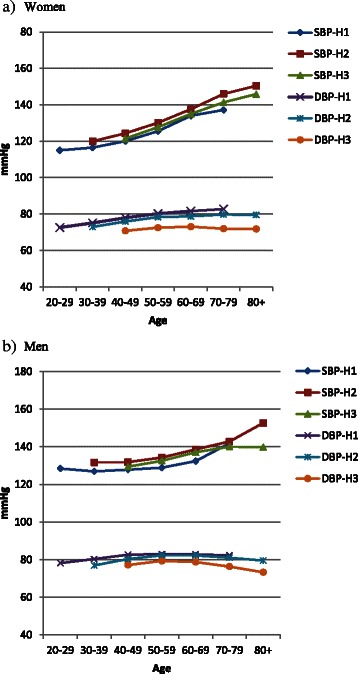
Individuals participating both in HUNT1 (H1), HUNT2 (H2) and HUNT3 (H3) and reporting never taking antihypertensive medication. Mean systolic (SBP) and diastolic (DBP) blood pressure in women (**a**, *n* = 8947) and men (**b**, *n* = 7414) by age. (Additional file 1: Table S5)

We next examined individuals participating in all three surveys and not reporting use of BP medication (8,947 women and 7,414 men). As expected, in both genders mean SBP increased from HUNT1 to HUNT2 (8.9 mmHg in women and 5.8 mmHg in men). The increase from HUNT2 to HUNT3 was less pronounced, and in one age group (60–79 years) the observed BP was lower than 11 years previously. Mean DBP was roughly identical in HUNT1 and HUNT2. However, from HUNT2 to HUNT3 the observed diastolic pressures showed a general *decrease* - despite that the participants were 11 years older, and the decrease was more pronounced in the older age groups in both sexes (Fig. 5, Additional file 1: Table S5).

### Predictors of blood pressure decrease

To follow up on this observation, we further examined the BP of participants not under current or past treatment. We found several changes in cardiovascular related risk factors from HUNT2 to HUNT3. Among these individuals both BMI and non-fasting glucose increased, while total cholesterol, heart rate and self-reported daily smoking decreased. Also, we observed that self-reported hard physical activity increased in the same period. These changes occurred in both sexes (Table 3). In bivariate analyses the mean BP decrease from HUNT2 to HUNT3 was most pronounced in individuals with the lowest level of education (both SBP and DBP; Additional file 1: Table S6). Also, the BP decrease was most pronounced among obese participants (BMI ≥ 30 kg/m^2^; Additional file 1: Table S7) and in those in the highest quartile of total serum cholesterol (Additional file 1: Table S8). Parallel to the BP decrease, we also observed a decrease in mean heart rate, from 76.4/73.7 beats/min in HUNT1 via 75.3/71.0 in HUNT2 to 71.9/68.3 beats/min in HUNT3 in women/men respectively (Additional file 1: Table S9).

**Table 3:** Participants who reported they had never taken blood pressure medication: Mean Body Mass Index (BMI), total serum cholesterol, non-fasting serum glucose, and heart rate in HUNT2 and HUNT3 (a), and prevalence (%) of self-reported daily smoking, hard physical activity and diabetes in HUNT2 and HUNT3 by gender (b)

		Women		Men	
(a)		Mean	SD	Mean	SD
BMI (kg/m^2^)	HUNT2^1^	25.89	4.30	26.22	3.37
	HUNT3^2^	26.34	4.62	27.07	3.64
Total cholesterol (mmol/l)	HUNT2	5.85	1.32	5.77	1.17
	HUNT3	5.34	1.14	5.48	1.06
Non fasting Se glucose (mmol/l)	HUNT2	5.27	1.23	5.47	1.50
	HUNT3	5.32	1.22	5.59	1.53
Heart rate (beats/min)	HUNT2	75.27	12.16	71.06	12.42
	HUNT3	71.87	11.03	68.34	11.31
(b)		%		%	
Self-reported daily smoking	HUNT2	31.5		29.7	
	HUNT3	20.3		15.7	
Self-reported hard physical activity	HUNT2	8.7		17.9	
(>3 h/week)	HUNT3	20.7		26.1	
Self-reported diabetes	HUNT2	1.7		2.3	
	HUNT3	2.1		2.6	

^1^ Number of individuals with valid measures in HUNT2 varied from 28,807 to 29,423 in women and from 26,223 and 26,509 in men, except the question about physical activity which was answered by 20,112 women and 20,393 men

^2^ Number of individuals with valid measures HUNT3 varied from 19,254 to 22,065 in women and between 15,814 and 18,114 men, except the question about physical activity which was answered by 12,511 women and 10,108 men

As a next step we analysed only those individuals who had participated both in HUNT1, HUNT2 and HUNT3 and reported they had never taken BP medication. While SBP had not changed substantially from HUNT2 to HUNT3 in these individuals, despite they were 11 years older, DBP had decreased substantially (Fig. 5, Additional file 1: Table S5). We therefore specified linear regression models with delta DBP (DBP in HUNT2 minus DBP in HUNT3) as the dependent variable, and found that the observed DBP decrease from HUNT2 to HUNT3 was associated with increasing age, decreasing BMI, decreasing total serum cholesterol and decreasing heart rate. These associations were consistent in both women and men. In men there was also an association with myocardial infarction (Table 4).

**Table 4:** Participants both in HUNT1, HUNT2 and HUNT3, reporting they had never used antihypertensive medication. Linear regression models with association between delta DBP (change in DBP from HUNT2 to HUNT3, i.e., DBP in HUNT2 minus DBP in HUNT3) and relevant confounders: Age, delta BMI (BMI in HUNT2 minus BMI at HUNT3), delta Se-cholesterol (total cholesterol at HUNT2 minus total cholesterol at HUNT3), delta heart rate (HR) (HR at HUNT2 minus HR at HUNT3), self-reported myocardial infarction (MI) (at HUNT3), delta smoking (pack years in HUNT2 minus pack years in HUNT3), self-reported diabetes (at HUNT3) (Yes = 1, No = 0) and education level (Low = 1, High = 5)

	Delta DBP			
	Women^1^		Men^2^	
	Beta	p	Beta	p
Age (years, at HUNT2)	.137	<.001	.126	<.001
Delta BMI (kg/m^2^)	.093	<.001	.137	<.001
Delta Se-cholesterol (mmol/l)	.107	<.001	.143	<.001
Delta HR (beats/min)	.169	<.001	.168	<.001
Self-reported MI (Yes = 1, No = 0)			.062	<.001

^1^Excluded from the model (not significant): Delta smoking, self-reported diabetes, non-fasting glucose, hard physical exercise, education level, self-reported MI

^2^Excluded from the model (not significant): Delta smoking, self-reported diabetes, non-fasting glucose, hard physical exercise, education level

## Discussion

In this large population based study, mean BP decreased during the whole period from mid 1980s (HUNT1) to mid 2000s (HUNT3), but most pronounced from mid 1990s (HUNT2) to mid 2000s (HUNT3). The decrease was observed both in SBP and DBP, in both genders and the decrease increased by age. The volume of antihypertensive drug treatment increased in the same period and explains some of the BP decrease. However, the BP decrease was significant even in those who reported they had never taken BP medication. An unexpected finding was the consistent association between blood pressure decrease with ageing and with decreasing heart rate.

Strengths of our study are the large unselected and stable population, the large number of health variables and the consistent methods for clinical measurements and data collection. In HUNT1 BP was measured manually, while in HUNT2 and HUNT3 it was used automatic devices. The devices were checked and calibrated by specialists several times during the examination periods, finding no major errors. The automatic method was calibrated against manually procedures, showing slightly lower values, but this could not explain the large discrepancies in BP levels between the HUNT2 and HUNT3 surveys [8]. Additionally, in HUNT2 and HUNT3 the same type of devices and the same protocol were used, and in all three surveys the measurements were performed by specially trained nurses, and the routines were checked repeatedly during the examination periods. Limits of the present study were the lower participation rate in HUNT3 compared to HUNT2 and HUNT1, especially in the age groups 20–39 and 80+ [6]. The comprehensive non-participation study in HUNT3 demonstrated that proportions of patients with diagnosis of cardiovascular diseases in general practitioner records was higher than the prevalence of corresponding self-reported diseases in HUNT3, but there was close agreement between the two data sets regarding use of antihypertensive treatment [7]. As a consequence, we have no reason to assume that selection bias was a major factor. Another limitation in the present study was the lack of information on salt and potassium consumption.

The increased drug treatment since HUNT2 was an important aspect when studying blood pressure changes in this population. We therefore estimated in a model the potential effect on population BP level from the increased drug treatment between HUNT2 and HUNT3: Provided those with BP above the 90 percentile in HUNT2 had their SBP decreased with 20 mmHg and DBP decreased with 10 mmHg, this effect on a population level could only explain about 2 mmHg decrease in SBP and 1 mmHg in DBP (data not shown).

The main challenge is to understand the large BP decrease from HUNT2 to HUNT3 in individuals reporting they had never taken BP medication, and especially since the decrease occurred in a period with large increase in body weight and diabetes. Recall bias or misinterpretation of the purpose of drugs prescribed might occur, however, validation of this question gave no indication of major misclassification [12]. The cardiovascular risk profile improved during the period, consistent with decreased cardiovascular morbidity and mortality in the population [13, 14]. Data on lipid lowering drugs were not available, but national statistics demonstrates that the consumption of these drugs had a large increase in the period from mid 1990s to mid 2000s [15]. There is, however, no evidence that lipid lowering drugs may have the effect on BP level demonstrated in the present study [16, 17].

BP decrease is reported in other industrialized populations since 1990s, along with increasing body weight [18]. In the WHO MONICA project covering 38 populations in 21 countries from mid 1980s to mid1990s average BP decreased 2.26 mmHg [2], while a study from England demonstrated that mean SBP had fallen by 1.6 and 4.3 mmHg in men and women in the period from 1994 to 2003 [19]. In the first study from Norway describing the BP decrease, including 40–42 year old participants from eight counties, demonstrated systolic decrease of 6.7 mmHg in women and 6.1 mmHg in men and diastolic decrease of 5.6 mmHg in women and 5.4 mmHg in men, without finding a reasonable explanation to the decrease [20]. A recent report from the Tromsø Study, Norway, found that mean systolic and diastolic pressure decreased from 1979 to 2008 in both genders in the age group 30 to 89 years. The decrease in systolic blood pressure in age group 40–49 years was 10.6 mmHg in women and 4.5 mmHg in men [21].

The larger BP decrease with increasing age in the present study has, to our knowledge, not been described previously. The parallel decrease in BP and heart rate and the association between BP decrease and heart rate decrease (linear regression) might indicate some kind of increased parasympathetic activity with strongest effect in elderly obese individuals. One explanation could be reduction in the white-coat phenomenon, but we have no data indicating why this effect should be so strong in the period from HUNT2 to HUNT3. Increased use of calcium channel blockers and beta blockers for other diagnosis than hypertension might explain some of the heart rate and blood pressure decrease, however, no data was available. In the period between HUNT2 and HUNT3 the Norwegian population increased consumption of fruit and vegetables, increasing the potassium (K) intake [22]. If the change in food habits also has induced less salt (NaCl) consumption, especially in elderly obese individuals, this might explain some of the BP decrease [23]. Considering the increased salt sensitivity in elderly [24] this might even explain the increasing BP decrease by age [25, 26]. Another hypothesis might be that subgroups in the population can sustain obesity without serious health consequences, i.e., the “healthy obese” hypothesis [27]. In another study of the same population, obesity without metabolic abnormalities did not confer substantial excess risk for myocardial infarction, a major cause of death [28]. Both the use of medication (other than for hypertension), the hypothesis about increased parasympathetic activity, the hypothesis about decreased salt consumption in elderly and the “healthy obese” hypothesis, should be explored in future studies.

## Conclusion

This large population based health study has demonstrated that in the period from mid 1980s to mid 2000s BP in the population decreased considerably, especially in the period from mid 1990s to mid 2000s, and even in those who reported they had never taken BP medication. The BP decrease was larger in elderly and might seem paradoxically given the fact that body weight and the prevalence of diabetes increased during the same period. The BP decrease was, however, consistent with the general improvement in the cardiovascular risk profile and decreasing cardiovascular morbidity and mortality. We have no reason to assume that the methods of blood pressure measurement or selection bias can explain the findings. The parallel decrease in heart rate might indicate increased parasympathetic activity, like reduction in the white-coat phenomenon, and a possible increased use of beta blockers or calcium channel blockers for other diagnosis than hypertension could have had some effect. Additionally, salt consumption might have decreased. Finally, the data could support the “healthy obese” hypothesis, i.e., that subgroups in the population can sustain obesity without serious health consequences.

### Ethics approval and consent to participate

The participation was voluntary and participants gave their written consent to participate and the use of data in research. All three surveys were approved by the Norwegian Data Inspectorate. In addition, HUNT2 and HUNT3 were approved by the Regional Committee for Medical and Health Research Ethics (REC). At the time of HUNT1 REC was not yet established. The current study protocol was approved separately by REC.

### Consent for publication

HUNT Research Centre, University of Science and Technology (NTNU), has consented to publish the present manuscript.

### Availability of data and materials

All HUNT-data are stored at HUNT Research Centre, and are available upon request according to general rules, see http://www.ntnu.edu/hunt.

## Additional file

Additional file 1: Supplementary tables and figures. (DOCX 97 kb)

## Abbreviations

BMI: body mass index
BP: blood pressure
DBP: diastolic blood pressure
HR: heart rate
HUNT/the HUNT Study: The Health Study of Nord-Trøndelag, Norway
HUNT1: The first survey of the HUNT Study (1984–86)
HUNT2: The second survey of the HUNT Study (1995–97)
HUNT3: The third survey of the HUNT Study (2006–08)
REC: The Regional Committee for Medical and Health Research Ethics
SBP: systolic blood pressure
WHO: World Health Organisation
