# Supplementary material for: Blood pressure changes during 22-year of follow-up in large general population - the HUNT Study, Norway
Source: BMC Cardiovasc Disord. 2016 May 12;16:94. doi: 10.1186/s12872-016-0257-8 (PMC4866289; doi:10.1186/s12872-016-0257-8)
Supplement: Additional file 1: — Supplementary tables and figures. (DOCX 97 kb) [file 12872_2016_257_MOESM1_ESM.docx]

**Supplementary Table** 1. Number of participants (n) and mean systolic (SBP) and diastolic blood pressure (DBP) with standard deviation (SD)

in HUNT1, HUNT2 and HUNT3 by age and gender.

1. Women

|  | HUNT 1 (1984-86) | | | | | HUNT 2 (1995-97) | | | | | HUNT 3 (2006-08) | | | | |
| --- | --- | --- | --- | --- | --- | --- | --- | --- | --- | --- | --- | --- | --- | --- | --- |
| Age | n | SBP mmHg | SD | DBP mmHg | SD | n | SBP mmHg | SD | DBP mmHg | SD | n | SBP mmHg | SD | DBP mmHg | SD |
| 20-29 | 5831 | 117.0 | 11.1 | 74.0 | 9.4 | 4783 | 120.6 | 11.5 | 70.5 | 8.5 | 2200 | 115.4 | 10.3 | 64.3 | 8.2 |
| 30-39 | 8008 | 119.6 | 13.3 | 77.6 | 9.6 | 6161 | 120.9 | 12.8 | 73.4 | 9.3 | 3511 | 117.2 | 12.0 | 67.8 | 9.3 |
| 40-49 | 6009 | 126.7 | 16.9 | 82.1 | 10.3 | 7046 | 127.9 | 16.7 | 78.2 | 10.5 | 4728 | 120.9 | 14.4 | 70.7 | 10.1 |
| 50-59 | 5643 | 138.4 | 21.8 | 86.4 | 11.2 | 5702 | 138.0 | 20.0 | 82.0 | 11.3 | 5214 | 129.0 | 17.5 | 73.3 | 10.4 |
| 60-69 | 6263 | 151.5 | 24.2 | 88.3 | 11.3 | 4689 | 149.6 | 22.6 | 83.8 | 12.3 | 4449 | 137.0 | 19.3 | 73.4 | 10.9 |
| 70-79 | 4635 | 161.0 | 25.7 | 88.7 | 12.0 | 4314 | 159.6 | 23.8 | 84.9 | 13.9 | 2647 | 142.4 | 21.1 | 72 | 11.7 |
| 80+ | 1784 | 163.2 | 28.7 | 86.3 | 13.5 | 1630 | 165.2 | 25.5 | 85.9 | 15.4 | 1219 | 146.1 | 23.0 | 71.5 | 12.7 |
| 20+ | 38,173 | 135.4 | 25.7 | 82.6 | 12.0 | 34,325 | 136.0 | 23.8 | 78.9 | 12.4 | 23,968 | 128.3 | 19.5 | 70.9 | 10.8 |
| 1. Men |  |  |  |  |  |  |  |  |  |  |  |  |  |  |  |
|  |  | | | | | | | | | | | | |  |  |
|  | HUNT 1 (1984-86) | | | | | HUNT 2 (1995-97) | | | | | HUNT 3 (2006-08) | | | | |
| Age | n | SBP  mmHg | SD | DBP  mmHg | SD | n | SBP  mmHg | SD | DBP  mmHg | SD | n | SBP  mmHg | SD | DBP  mmHg | SD |
| 20-29 | 5857 | 129.8 | 12.8 | 79.3 | 9.5 | 3983 | 132.6 | 12.1 | 72.4 | 9.0 | 1525 | 126.0 | 11.2 | 65.6 | 8.9 |
| 30-39 | 7983 | 130.0 | 13.2 | 82.8 | 9.7 | 5386 | 132.6 | 12.6 | 77.5 | 9.3 | 2516 | 127.9 | 12.0 | 72.4 | 9.5 |
| 40-49 | 5954 | 133.0 | 15.3 | 86.5 | 10.2 | 6501 | 135.0 | 14.7 | 82.9 | 10.3 | 4011 | 129.5 | 13.4 | 76.8 | 10.0 |
| 50-59 | 5619 | 139.7 | 19.3 | 88.7 | 11.0 | 5318 | 141.0 | 18.0 | 86.3 | 10.8 | 4702 | 133.7 | 16.3 | 80.1 | 10.2 |
| 60-69 | 6069 | 147.7 | 22.5 | 88.9 | 11.4 | 4309 | 148.5 | 21.6 | 86.8 | 12.1 | 4051 | 138.7 | 18.6 | 79.4 | 10.5 |
| 70-79 | 3883 | 154.6 | 24.1 | 88.7 | 12.1 | 3613 | 153.2 | 22.7 | 85.6 | 12.7 | 2293 | 140.9 | 19.1 | 76.3 | 10.8 |
| 80+ | 1311 | 154.4 | 26.2 | 86.3 | 13.2 | 1088 | 155.7 | 25.3 | 84.2 | 13.6 | 833 | 140.8 | 22.5 | 73.2 | 11.7 |
| 20+ | 36,376 | 138.3 | 20.3 | 85.5 | 11.2 | 30,198 | 140.1 | 19.1 | 82.1 | 11.8 | 19,937 | 133.7 | 16.9 | 76.5 | 10.9 |

**Supplementary Table 2**. Blood pressure (BP) distribution in HUNT1, HUNT2 and HUNT3 according to the ESH/ESC definitions and classification

of office BP [11] by age: Optimal: SBP <120 mmHg and DBP <80 mmHg. Normal: SBP 120-129 mmHg and/or DBP 80-84 mmHg.

High normal: SBP 130-139 mmHg and/or DBP 85-89 mmHg. Hypertension (HT) Grade 1: SBP 140-159 mmHg and/or DBP 90-99 mmHg.

HT Grade 2: SBP 160-179 mmHg and/or 100-109 mmHg. HT Grade 3: SBP > 180 mmHg and/or DBP > 100 mmHg.

| 1. Women |  |  | | | | | | |
| --- | --- | --- | --- | --- | --- | --- | --- | --- |
|  |  |  | Optimal | Normal | High normal | HT Grade 1 | HT Grade 2 | HT Grade 3 |
| Age | Survey | n | % | % | % | % | % | % |
| 20-29 | HUNT1 | 5831 | 50.1 | 30.4 | 11.4 | 7.4 | 0.8 | 0.0 |
|  | HUNT2 | 4783 | 47.8 | 29.9 | 15.7 | 6.2 | 0.4 | 0.0 |
|  | HUNT3 | 2200 | 68.0 | 22.7 | 7.2 | 2.0 | 0.1 | 0.0 |
| 30-39 | HUNT1 | 8007 | 40.9 | 29.8 | 14.0 | 12.7 | 2.3 | 0.2 |
|  | HUNT2 | 6161 | 48.4 | 26.7 | 15.4 | 8.1 | 1.3 | 0.0 |
|  | HUNT3 | 3512 | 62.6 | 22.9 | 9.8 | 4.2 | 0.5 | 0.1 |
| 40-49 | HUNT1 | 6009 | 24.9 | 28.1 | 17.2 | 21.8 | 7.5 | 0.4 |
|  | HUNT2 | 7046 | 31.6 | 25.4 | 18.9 | 18.3 | 5.4 | 0.4 |
|  | HUNT3 | 4727 | 50.5 | 24.2 | 14.7 | 8.9 | 1.6 | 0.0 |
| 50-59 | HUNT1 | 5643 | 12.7 | 17.2 | 17.0 | 34.1 | 17.3 | 1.5 |
|  | HUNT2 | 5702 | 16.4 | 18.0 | 20.2 | 30.4 | 14.0 | 0.9 |
|  | HUNT3 | 5214 | 31.6 | 23.0 | 19.2 | 21.6 | 4.5 | 0.2 |
| 60-69 | HUNT1 | 6263 | 4.3 | 10.3 | 14.3 | 39.2 | 28.8 | 3.1 |
|  | HUNT2 | 4689 | 7.8 | 11.8 | 16.1 | 36.3 | 25.4 | 2.6 |
|  | HUNT3 | 4449 | 18.4 | 18.3 | 22.2 | 30.9 | 9.6 | 0.6 |
| 70-79 | HUNT1 | 4635 | 2.2 | 7.2 | 10.9 | 38.3 | 36.5 | 4.9 |
|  | HUNT2 | 4314 | 3.8 | 6.7 | 13.1 | 37.6 | 33.5 | 5.6 |
|  | HUNT3 | 2648 | 11.7 | 17.0 | 21.0 | 34.2 | 14.8 | 1.3 |
| 80+ | HUNT1 | 1784 | 3.5 | 7.3 | 11.6 | 37.6 | 34.4 | 5.5 |
|  | HUNT2 | 1630 | 2.6 | 7.3 | 13.4 | 33.0 | 34.3 | 9.4 |
|  | HUNT3 | 1219 | 11.9 | 12.0 | 17.8 | 37.8 | 18.0 | 2.5 |
| 20+ | HUNT1 | 38172 | 23.2 | 20.8 | 14.1 | 25.1 | 15.1 | 1.7 |
|  | HUNT2 | 34325 | 26.2 | 20.0 | 16.7 | 22.4 | 13.0 | 1.7 |
|  | HUNT3 | 23969 | 37.5 | 21.1 | 16.5 | 18.7 | 5.7 | 0.4 |

1. Men

|  |  |  | | | | | |  |
| --- | --- | --- | --- | --- | --- | --- | --- | --- |
|  |  |  | Optimal | Normal | High normal | HT Grade 1 | HT Grade 2 | HT Grade 3 |
| Age | Survey | n | % | % | % | % | % | % |
| 20-29 | HUNT1 | 5857 | 14.1 | 29.7 | 26.2 | 26.1 | 3.9 | 0.0 |
|  | HUNT2 | 3983 | 16.6 | 26.3 | 32.3 | 25.7 | 2.1 | 0.0 |
|  | HUNT3 | 1525 | 27.8 | 35.8 | 24.7 | 11.3 | 0.3 | 0.1 |
| 30-39 | HUNT1 | 7983 | 12.4 | 27.9 | 25.5 | 27.6 | 6.5 | 0.1 |
|  | HUNT2 | 5386 | 14.0 | 26.2 | 31.4 | 25.1 | 3.3 | 0.1 |
|  | HUNT3 | 2517 | 24.2 | 34.1 | 25.4 | 14.8 | 1.3 | 0.1 |
| 40-49 | HUNT1 | 5952 | 9.3 | 21.9 | 21.4 | 35.1 | 11.8 | 0.5 |
|  | HUNT2 | 6501 | 11.5 | 22.1 | 27.4 | 30.3 | 8.3 | 0.4 |
|  | HUNT3 | 4011 | 21.5 | 29.9 | 26.4 | 19.0 | 3.1 | 0.1 |
| 50-59 | HUNT1 | 5619 | 6.5 | 16.4 | 17.0 | 38.7 | 19.7 | 1.7 |
|  | HUNT2 | 5318 | 8.4 | 15.2 | 21.6 | 36.9 | 16.5 | 1.4 |
|  | HUNT3 | 4702 | 17.4 | 22.9 | 24.0 | 28.9 | 6.5 | 0.5 |
| 60-69 | HUNT1 | 6069 | 4.3 | 11.6 | 15.5 | 39.7 | 26.0 | 3.0 |
|  | HUNT2 | 4309 | 6.7 | 10.4 | 17.1 | 37.0 | 26.2 | 2.7 |
|  | HUNT3 | 4053 | 13.4 | 17.8 | 23.0 | 32.6 | 12.6 | 0.6 |
| 70-79 | HUNT1 | 3883 | 2.9 | 8.4 | 12.9 | 39.2 | 33.0 | 3.5 |
|  | HUNT2 | 3613 | 5.8 | 8.2 | 14.5 | 36.6 | 31.5 | 3.3 |
|  | HUNT3 | 2291 | 11.6 | 15.8 | 21.3 | 37.0 | 13.9 | 0.4 |
| 80+ | HUNT1 | 1311 | 5.9 | 11.5 | 11.1 | 36.5 | 31.1 | 4.0 |
|  | HUNT2 | 1088 | 5.8 | 9.2 | 14.5 | 37.7 | 28.0 | 4.8 |
|  | HUNT3 | 831 | 16.2 | 15.4 | 20.6 | 31.2 | 15.8 | 0.8 |
| 20+ | HUNT1 | 36674 | 8.7 | 20.1 | 20.1 | 33.8 | 15.9 | 1.4 |
|  | HUNT2 | 30198 | 10.1 | 18.4 | 24.3 | 31.9 | 14.0 | 1.3 |
|  | HUNT3 | 19930 | 18.3 | 24.6 | 24.1 | 25.6 | 7.2 | 0.3 |

**Supplementary Table 3.** Participants (n and %) with SBP >140 mmHg or DBP >90 mmHg and with SBP <140 mmHg and DBP <90 mmHg in HUNT1 (a), HUNT2 (b) and HUNT3 (c) by age groups and gender.

1. HUNT1

|  | Women | | | | Men | | | | Total | | | | |
| --- | --- | --- | --- | --- | --- | --- | --- | --- | --- | --- | --- | --- | --- |
|  | SBP>140 or DBP>90 | | SBP<140 and DBP<90 | | SBP>140 or DBP>90 | | SBP<140 and DBP<90 | | SBP>140 or DBP>90 | | SBP<140 and DBP<90 | | |
| Age | n | % | n | % | n | % | n | % | n | % | n | | % |
| 20-29 | 479 | 8.2 | 5352 | 91.8 | 1760 | 30.0 | 4097 | 70.0 | 2239 | 19.2 | 9449 | | 80.8 |
| 30-39 | 1225 | 15.3 | 6780 | 84.7 | 2731 | 34.2 | 5251 | 65.8 | 3956 | 24.7 | 12031 | | 75.3 |
| 40-49 | 1791 | 29.8 | 4218 | 70.2 | 2831 | 47.6 | 3121 | 52.4 | 4622 | 38.6 | 7339 | | 61.4 |
| 50-59 | 3018 | 53.5 | 2625 | 46.5 | 3396 | 60.4 | 2222 | 39.6 | 6414 | 57.0 | 4847 | | 43.0 |
| 60-69 | 4551 | 72.7 | 1711 | 27.3 | 4230 | 69.7 | 1839 | 30.3 | 8781 | 71.2 | 3550 | | 28.8 |
| 70-79 | 3855 | 83.2 | 780 | 16.8 | 3014 | 77.6 | 869 | 22.4 | 6869 | 80.6 | 1649 | | 19.4 |
| 80+ | 1475 | 82.7 | 309 | 17.3 | 985 | 75.1 | 326 | 24.2 | 2460 | 79.5 | 635 | | 20.5 |
| 20+ | 16394 | 43.0 | 21775 | 57.0 | 18947 | 51.7 | 17725 | 48.3 | 35341 | 47.2 | 39500 | | 52.8 |
| b) HUNT2 |  |  |  |  |  |  |  |  |  |  |  | |  |
|  | Women | | | | Men | | | | Both sexes | | | | |
|  | SBP>140 or DBP>90 | | SBP<140 and DBP<90 | | SBP>140 or DBP>90 | | SBP<140 and DBP<90 | | SBP>140 or DBP>90 | | SBP<140 and DBP<90 | | |
| Age | n | % | n | % | n | % | n | % | n | % | n | | % |
| 20-29 | 316 | 6.6 | 4467 | 93.4 | 1106 | 27.8 | 2877 | 72.2 | 1422 | 16.2 | 7344 | | 83.8 |
| 30-39 | 583 | 9.5 | 5578 | 90.5 | 1531 | 28.4 | 3855 | 71.6 | 2114 | 18.3 | 9433 | | 81.7 |
| 40-49 | 1701 | 24.1 | 5345 | 75.9 | 2538 | 39.0 | 3963 | 61.0 | 4239 | 31.3 | 9308 | | 68.7 |
| 50-59 | 2597 | 45.5 | 3105 | 54.5 | 2913 | 54.8 | 2405 | 45.2 | 5510 | 50.00 | 5510 | | 50.0 |
| 60-69 | 3087 | 65.8 | 1602 | 34.2 | 2872 | 66.7 | 1437 | 33.3 | 5959 | 66.2 | 3039 | | 33.8 |
| 70-79 | 3453 | 80.0 | 861 | 20.0 | 2651 | 73.4 | 962 | 26.6 | 6104 | 77.0 | 1823 | | 23.0 |
| 80+ | 1350 | 82.8 | 280 | 17.2 | 808 | 74.3 | 280 | 25.7 | 2158 | 79.4 | 560 | | 20.6 |
| 20+ | 13087 | 38.1 | 21238 | 61.9 | 14419 | 47.7 | 15779 | 52.3 | 27506 | 42.6 | 37017 | | 57.4 |
|  |  |  |  |  |  |  |  |  |  |  |  | |  |
| c)  HUNT3 |  |  |  |  |  |  |  |  |  |  |  | |  |
|  | Women | | | | Men | | | | Both sexes | | | | |
|  | SBP>140 or DBP>90 | | SBP<140 og DBP<90 | | SBP>140 or DBP>90 | | SBP<140 og DBP<90 | | SBP>140 or DBP>90 | | SBP<140 og DBP<90 | | |
| Age | n | % | n | % | n | % | n | % | n | % | n | % | |
| 20-29 | 47 | 2.1 | 2153 | 97.9 | 178 | 11.7 | 1347 | 88.3 | 225 | 6.0 | 3500 | 94.0 | |
| 30-39 | 167 | 4.8 | 3344 | 95.2 | 410 | 16.3 | 2106 | 83.7 | 577 | 9.6 | 5450 | 90.4 | |
| 40-49 | 503 | 10.6 | 4224 | 89.4 | 892 | 22.2 | 3119 | 77.8 | 1395 | 16.0 | 7343 | 84.0 | |
| 50-59 | 1384 | 26.5 | 3829 | 73.5 | 1681 | 35.8 | 3021 | 64.2 | 3065 | 30.9 | 6850 | 69.1 | |
| 60-69 | 1864 | 41.9 | 2585 | 58.1 | 1869 | 46.1 | 2184 | 53.9 | 3733 | 43.9 | 4769 | 56.1 | |
| 70-79 | 1384 | 52.3 | 1262 | 47.7 | 1197 | 52.2 | 1094 | 47.8 | 2581 | 52.3 | 2356 | 47.7 | |
| 80+ | 738 | 60.5 | 481 | 39.5 | 410 | 49.3 | 421 | 50.7 | 1148 | 56.0 | 902 | 44.0 | |
| 20+ | 6087 | 25.4 | 17878 | 74.6 | 6637 | 33.3 | 13292 | 66.7 | 12724 | 29.0 | 31170 | 71.0 | |

**Supplementary Table 4**. Participants reporting never taking blood pressure medication: Number of participants (n) and mean systolic (SBP) and diastolic (DBP) blood pressure in HUNT1. HUNT2 and HUNT3 by age. Differences between SBP and DBP in HUNT2 and HUNT 3 (Delta) in each age group.

a)Women

| Women | HUNT 1 | | | | | HUNT 2 | | | | | HUNT 3 | | | | | Delta BP  (HUNT 2-HUNT3) | |
| --- | --- | --- | --- | --- | --- | --- | --- | --- | --- | --- | --- | --- | --- | --- | --- | --- | --- |
| Age | n | SBP |  | DBP |  | n | SBP |  | DBP |  | n | SBP |  | DBP |  | *Delta SBP* | *Delta DBP* |
|  |  | mmHg | SD | mmHg | SD |  | mmHg | SD | mmHg | SD |  | mmHg | SD | mmHg | SD | *mmHg* | *mmHg* |
| 20-29 | 5731 | 116.8 | 11.0 | 73.9 | 9.3 | 4712 | 120.5 | 11.3 | 70.4 | 8.4 | 2176 | 115.4 | 10.2 | 64.2 | 8.2 | *5.1* | *6.2* |
| 30-39 | 7776 | 119.2 | 12.9 | 77.2 | 9.4 | 5964 | 120.5 | 12.5 | 73.1 | 9.1 | 3406 | 116.8 | 11.7 | 67.6 | 9.1 | *3.7* | *5.5* |
| 40-49 | 5649 | 125.7 | 16.2 | 81.4 | 9.8 | 6646 | 126.9 | 16.0 | 77.6 | 10.1 | 4379 | 120.1 | 14.0 | 70.2 | 9.9 | *6.8* | *7.4* |
| 50-59 | 4659 | 135.5 | 20.8 | 84.9 | 10.8 | 4869 | 136.1 | 19.4 | 81.0 | 10.9 | 4235 | 127.3 | 17.1 | 72.5 | 10.3 | *8.8* | *8.5* |
| 60-69 | 4293 | 147.4 | 23.4 | 86.3 | 11.1 | 3391 | 146.7 | 22.4 | 82.4 | 12.0 | 2978 | 135.5 | 19.2 | 73.0 | 11.0 | *11.2* | *9.4* |
| 70-79 | 2798 | 156.7 | 25.3 | 86.4 | 11.9 | 2600 | 155.7 | 23.8 | 83.2 | 13.5 | 1346 | 141.6 | 21.3 | 72.1 | 11.5 | *14.1* | *11.1* |
| 80+ | 1208 | 159.9 | 27.9 | 84.5 | 13.1 | 996 | 161.9 | 24.9 | 84.1 | 15.0 | 586 | 145.8 | 22.8 | 71.7 | 12.3 | *16.1* | *12.4* |
| 20+ | 32,114 | 130.9 | 23.1 | 80.8 | 11.3 | 29178 | 132.1 | 21.6 | 77.4 | 11.5 | 19106 | 125.3 | 18.2 | 70.2 | 10.4 | *6.8* | *7.2* |
|  |  |  |  |  |  |  |  |  |  |  |  |  |  |  |  |  |  |
| b)Men |  |  |  |  |  |  |  |  |  |  |  |  |  |  |  |  |  |
| Men | HUNT 1 | | | | | HUNT 2 | | | | | HUNT 3 | | | | | Delta BP  (HUNT 2-HUNT3) | |
| Age | n | SBP |  | DBP |  | n | SBP |  | DBP |  | n | SBP |  | DBP |  | *Delta SBP* | *Delta DBP* |
|  |  | mmHg | SD | mmHg | SD |  | mmHg | SD | mmHg | SD |  | mmHg | SD | mmHg | SD | *mmHg* | *mmHg* |
| 20-29 | 5822 | 129.8 | 12.7 | 79.2 | 9.5 | 3951 | 132.6 | 12.0 | 72.4 | 8.9 | 1514 | 125.9 | 11.0 | 65.5 | 8.8 | *6.7* | *6.9* |
| 30-39 | 7792 | 129.7 | 13 | 82.5 | 9.4 | 5277 | 132.3 | 12.5 | 77.3 | 9.2 | 2465 | 127.6 | 11.6 | 72.2 | 9.3 | *4.7* | *5.1* |
| 40-49 | 5610 | 132.3 | 14.8 | 85.9 | 9.8 | 6091 | 134.2 | 14.1 | 82.3 | 9.9 | 3694 | 128.8 | 13.0 | 76.3 | 9.8 | *5.4* | *6,0* |
| 50-59 | 4869 | 138.2 | 18.7 | 87.6 | 10.6 | 4526 | 139.3 | 17.2 | 85.3 | 10.4 | 3757 | 132.5 | 15.9 | 79.4 | 10.0 | *6.8* | *5.9* |
| 60-69 | 4789 | 145.2 | 21.4 | 87.4 | 11.0 | 3193 | 146.3 | 20.7 | 85.7 | 11.6 | 2630 | 137.1 | 19.2 | 78.8 | 10.5 | *9.2* | *6.9* |
| 70-79 | 3017 | 152.0 | 23.4 | 87.0 | 11.7 | 2524 | 150.6 | 22.0 | 84.3 | 12.3 | 1198 | 140.0 | 19.3 | 76.2 | 10.6 | *10.6* | *8.1* |
| 80+ | 1101 | 152.6 | 25.9 | 85.3 | 12.8 | 788 | 154.2 | 25.3 | 83.6 | 13.4 | 434 | 139.5 | 23.4 | 73.2 | 12.2 | *14.7* | *10.4* |
| 20+ | 33,000 | 136.5 | 19 | 84.5 | 10.7 | 26350 | 138.1 | 17.7 | 81.0 | 11.3 | 15692 | 131.8 | 15.9 | 75.7 | 10.7 | *6.3* | *5.3* |

| **Supplementary Table 5. I**ndividuals participating both in HUNT1, HUNT2 and HUNT3 and reporting that they had never used antihypertensive medication. Number of individuals (n) and mean SBP (a) and DBP (b) by age.   1. SBP | | | | | | | | | | |  |  |
| --- | --- | --- | --- | --- | --- | --- | --- | --- | --- | --- | --- | --- |
|  | Women | | | | | | Men | | | | | |
|  | HUNT1 | | HUNT2 | | HUNT3 | | HUNT1 | | HUNT2 | | HUNT3 | |
| Age | n | SBP mmHg | n | SBP  mmHg | n | SBP  mmHg | n | SBP  mmHg | n | SBP  mmHg | n | SBP  mmHg |
| 20-29 | 2348 | 115.0 |  |  |  |  | 1881 | 128.4 |  |  |  |  |
| 30-39 | 3250 | 116.5 | 1935 | 119.90 |  |  | 2730 | 126.9 | 1539 | 131.6 |  |  |
| 40-49 | 2062 | 120.0 | 3222 | 124.30 | 1586 | 121.4 | 1742 | 127.8 | 2714 | 131.8 | 1278 | 129.4 |
| 50-59 | 1003 | 125.5 | 2317 | 130.10 | 3164 | 127.7 | 835 | 128.9 | 1931 | 134.3 | 2660 | 132.5 |
| 60-69 | 272 | 134.0 | 1081 | 137.60 | 2535 | 135.0 | 217 | 132.4 | 924 | 138.6 | 2094 | 137.1 |
| 70-79 | 12 | 137.2 | 368 | 145.96 | 1176 | 141.3 | 9 | 141.8 | 294 | 142.8 | 1007 | 140.0 |
| 80+ |  |  | 24 | 150.40 | 486 | 145.9 |  |  | 12 | 152.6 | 375 | 139.8 |
| 20+ | 8947 | 118.5 | 8947 | 127.40 | 8947 | 131.6 | 7414 | 127.9 | 7414 | 133.8 | 7414 | 134.7 |
| 1. DBP |  |  |  |  |  |  |  |  |  |  |  |  |
|  |  |  |  |  |  |  |  |  |  |  |  |  |
|  | Women | | | | | | Men | | | | | |
|  | HUNT1 | | HUNT2 | | HUNT3 | | HUNT1 | | HUNT2 | | HUNT3 | |
| Age | n | DBP  mmHg | n | DBP  mmHg | n | DBP  mmHg | n | DBP  mmHg | n | DBP  mmHg | n | DBP  mmHg |
| 20-29 | 2348 | 72.6 |  |  |  |  | 1881 | 78.1 |  |  |  |  |
| 30-39 | 3249 | 75.1 | 1935 | 72.90 |  |  | 2729 | 80.3 | 1539 | 76.9 |  |  |
| 40-49 | 2062 | 77.9 | 3222 | 75.90 | 1586 | 70.8 | 1742 | 82.6 | 2714 | 80.3 | 1278 | 77.1 |
| 50-59 | 1003 | 80.2 | 2317 | 78.30 | 3164 | 72.5 | 835 | 83.0 | 1931 | 82.3 | 2659 | 79.3 |
| 60-69 | 272 | 81.6 | 1081 | 78.70 | 2534 | 73.1 | 217 | 82.8 | 924 | 82.2 | 2094 | 78.7 |
| 70-79 | 12 | 82.7 | 368 | 79.70 | 1175 | 72.0 | 9 | 82.2 | 294 | 81.0 | 1007 | 76.3 |
| 80+ |  |  | 24 | 79.50 | 486 | 71.8 |  |  | 24 | 79.5 | 375 | 73.3 |
| 20+ | 8946 | 75.9 | 8947 | 76.40 | 8945 | 72.2 | 7413 | 80.7 | 7414 | 80.4 | 7413 | 78.1 |

**Supplementary Table 6.** Participants reporting never taking blood pressure medication: Mean systolic (SBP) and diastolic (DBP) in HUNT2 and HUNT3 by self-reported level of education (at HUNT2), and differences (Delta) between SBP and DBP in HUNT 2 and HUNT3 in each category: Level I: Primary school 7-10 years, continuation school, folk high school. Level II: High school, intermediate school, vocational school, 1-2 years high school. Level III: University qualifying examination, junior college, A levels. Level IV: University and other post-secondary education, less than 4 years. Level V: University/college, 4 years or more.

1. Women

| Level of | SBP HUNT2 | | | SBP HUNT3 | | | *Delta SBP* | DBP HUNT2 | | DBP HUNT3 | | *Delta DBP* |
| --- | --- | --- | --- | --- | --- | --- | --- | --- | --- | --- | --- | --- |
| education | N | mmHg | SD |  | mmHg | SD | *mmHg* | mmHg | SD | mmHg | SD | *mmHg* |
| I (low) | 10026 | 141.6 | 23.6 | 3709 | 135.6 | 20.6 | *6.0* | 80.9 | 12.0 | 72.9 | 11.1 | *8.0* |
| II | 8470 | 127.8 | 18.0 | 4414 | 126.9 | 18.2 | *0.9* | 76.2 | 10.8 | 71.4 | 10.5 | *4.8* |
| III | 3331 | 122.7 | 14.6 | 1617 | 122.0 | 15.3 | *0.7* | 72.9 | 9.6 | 70.1 | 9.8 | *2.8* |
| IV | 3946 | 123.6 | 14.9 | 2173 | 123.1 | 16.3 | *0.5* | 73.9 | 9.7 | 70.1 | 9.8 | *3.8* |
| V (high) | 2170 | 123.7 | 16.1 | 1209 | 124.5 | 15.9 | *-0.8* | 74.5 | 10.0 | 70.3 | 10.1 | *4.2* |
|  |  |  |  |  |  |  |  |  |  |  |  |  |
| 1. Men |  |  |  |  |  |  |  |  |  |  |  |  |
|  |  |  |  |  |  |  |  |  |  |  |  |  |
|  |  |  | | | | |  |  | | | |  |
| Level of | SBP HUNT2 | | | SBP HUNT3 | | | *Delta SBP* | DBP HUNT2 | | DBP HUNT3 | | *Delta DBP* |
| education | N | mmHg | SD | n | mmHg | SD | *mmHg* | mmHg | SD | mmHg | SD | *mmHg* |
| I (low) | 7577 | 142.8 | 19.8 | 2440 | 135.7 | 18.6 | *7.1* | 83.6 | 11.5 | 77.6 | 10.8 | *6.0* |
| II | 10395 | 136.5 | 16.1 | 4731 | 132.8 | 15.9 | *3.7* | 79.9 | 10.9 | 77.2 | 10.1 | *2.7* |
| III | 2170 | 134.1 | 14.9 | 913 | 130.4 | 14.2 | *3.7* | 76.8 | 11.3 | 76.1 | 10.6 | *0.7* |
| IV | 3054 | 134.5 | 14.9 | 1443 | 132.4 | 15.5 | *2.1* | 79.6 | 10.7 | 77.4 | 10.1 | *2.2* |
| V (high) | 2186 | 134.2 | 15.5 | 1102 | 131.8 | 15.9 | *2.4* | 80.6 | 10.4 | 76.8 | 10.0 | *3.8* |
|  |  |  |  |  |  |  |  |  |  |  |  |  |

**Supplementary Table 7**. Participants reporting never taking blood pressure medication: Mean systolic (SBP) and diastolic (DBP)

blood pressure in HUNT2 and HUNT3 by categories of Body Mass Index (BMI), and differences (Delta) between SBP and DBP

in HUNT 2 and HUNT3 in each category.

1. Women

|  | SBP HUNT2 | | | SBP HUNT3 | | | *Delta* | DBP HUNT2 | | DBP HUNT3 | | Delta |
| --- | --- | --- | --- | --- | --- | --- | --- | --- | --- | --- | --- | --- |
| BMI (kg/m^2^) | N | mmHg | SD | n | mmHg | SD | *mmHg* | mmHg | SD | mmHg | SD | *mmHg* |
| <18.5 | 321 | 127.8 | 22.6 | 184 | 119.5 | 20.1 | *8.3* | 74.6 | 11.9 | 67.3 | 11.4 | *7.3* |
| 18.5-24.9 | 13682 | 126.4 | 19.1 | 8232 | 121.0 | 17.1 | *5.4* | 74.6 | 10.5 | 68.2 | 10.2 | *6.4* |
| 25.0-29.9 | 10627 | 135.0 | 21.5 | 7094 | 127.0 | 18.0 | *8.0* | 78.8 | 11.5 | 71.0 | 10.3 | *7.8* |
| >30 | 4433 | 142.4 | 23.1 | 3628 | 131.6 | 18.1 | *10.8* | 82.3 | 12.2 | 73.0 | 10.3 | *9.3* |
|  |  |  |  |  |  |  |  |  |  |  |  |  |
| 1. Men |  |  |  |  |  |  |  |  |  |  |  |  |
|  |  |  | | | | |  |  | | | |  |
|  | SBP HUNT2 | | | SBP HUNT3 | | | *Delta* | DBP HUNT2 | | DBP HUNT3 | | Delta |
| BMI (kg/m^2^) | N | mmHg | SD | n | mmHg | SD | *mmHg* | mmHg | SD | mmHg | SD | *mmHg* |
| <18.5 | 107 | 130.8 | 18.3 | 60 | 124.9 | 18.3 | *5.9* | 73.8 | 10.8 | 71.6 | 12.0 | *2.2* |
| 18.5-24.9 | 9711 | 134.5 | 17.0 | 4397 | 127.7 | 15.6 | *6.8* | 77.8 | 10.8 | 72.1 | 10.5 | *5.7* |
| 25.0-29.9 | 13230 | 139.4 | 17.5 | 8349 | 132.4 | 15.5 | *7.0* | 82.2 | 11.0 | 76.4 | 10.5 | *5.8* |
| >30 | 3306 | 143.1 | 18.2 | 2925 | 136.1 | 15.9 | *7.0* | 85.1 | 11.7 | 78.8 | 10.6 | *6.3* |

**Supplementary Table 8.** Participants reporting never taking blood pressure medication: Mean systolic (SBP) and diastolic (DBP)

blood pressure in HUNT2 and HUNT3 by categories of total serum cholesterol, (quartiles, Q1-Q4, by HUNT2) and differences

(Delta) between SBP and DBP in HUNT 2 and HUNT3 in each category.

1. Women

| Total cholesterol | SBP HUNT2 | | | SBP HUNT3 | | | *Delta* | DBP HUNT2 | | DBP HUNT3 | | *Delta* |
| --- | --- | --- | --- | --- | --- | --- | --- | --- | --- | --- | --- | --- |
| (mmol/l) | N | mmHg | SD | n | mmHg | SD | *mmHg* | mmHg | SD | mmHg | SD | *mmHg* |
| Q1 (<4.8) | 6148 | 121.0 | 14.1 | 3240 | 120.9 | 15.1 | *0.1* | 71.2 | 9.3 | 69.6 | 9.7 | *1.6* |
| Q2 (4.8-5.59) | 7109 | 126.7 | 17.6 | 3619 | 125.8 | 17.7 | *0.9* | 75.4 | 10.2 | 71.0 | 10.3 | *4.4* |
| Q3 (5.6-6.4) | 7109 | 132.9 | 20.8 | 2994 | 129.7 | 18.9 | *3.2* | 78.2 | 11.4 | 72.1 | 10.7 | *6.1* |
| Q4 (>6.4) | 9431 | 142.9 | 23.7 | 3504 | 136 | 20.8 | *6.9* | 81.8 | 12.1 | 72.8 | 11.0 | *9.0* |
|  | 29234 | 132.1 | 21.6 | 13357 | 128.2 | 19.1 | *3.9* | 77.3 | 11.5 | 71.4 | 10.5 | 5.9 |
|  |  |  |  |  |  |  |  |  |  |  |  |  |
| 1. Men |  |  |  |  |  |  |  |  |  |  |  |  |
|  |  | | | | | | |  | | | | |
| Total cholesterol | SBP HUNT2 | | | SBP HUNT3 | | | *Delta* | DBP HUNT2 | | DBP HUNT3 | | *Delta* |
| (mmol/l) | N | mmHg | SD | n | mmHg | SD | *mmHg* | mmHg | SD | mmHg | SD | *mmHg* |
| Q1 (<4.8) | 5045 | 132.7 | 14.8 | 2077 | 129.3 | 14.4 | *3.4* | 75.2 | 10.6 | 75.0 | 10.0 | *0.2* |
| Q2 (4.8-5.59) | 6612 | 136.3 | 17.0 | 2876 | 132.1 | 16.0 | *4.2* | 79.4 | 10.7 | 76.9 | 10 | *2.5* |
| Q3 (5.6-6.4) | 6986 | 139.3 | 17.9 | 2931 | 134.4 | 17.0 | *4.9* | 82.3 | 10.9 | 77.9 | 10.5 | *4.4* |
| Q4 (>6.4) | 7758 | 142 | 18.7 | 2897 | 135.8 | 17.7 | *6.2* | 84.6 | 10.9 | 78.4 | 10.4 | *6.2* |
|  | 26401 | 138.1 | 17.7 | 10781 | 133.2 | 16.6 | *4.9* | 80.9 | 11.3 | 77.2 | 10.3 | *3.7* |

**Supplementary Table 9**. Participants reporting never taking blood pressure medication: Mean heart rate (beats/minute)

in HUNT1, HUNT2 and HUNT2 by age.

1. Women

|  | HUNT1 | | | HUNT2 | | | HUNT3 | | |
| --- | --- | --- | --- | --- | --- | --- | --- | --- | --- |
|  |  | Heart rate | |  | Heart rate | |  | Heart rate | |
| Age | N | Beats/min | SD | n | Beats/min | SD | n | Beats/min | SD |
| 20-29 | 5734 | 75.6 | 11.8 | 4711 | 75.7 | 12.0 | 2177 | 73.7 | 11.1 |
| 30-39 | 7782 | 75.9 | 11.8 | 5962 | 74.8 | 11.6 | 3413 | 71.9 | 10.6 |
| 40-49 | 5649 | 75.8 | 11.8 | 6644 | 74.9 | 12.0 | 4392 | 70.9 | 10.6 |
| 50-59 | 4659 | 75.9 | 12.1 | 4867 | 74.7 | 12.2 | 4249 | 71.3 | 11.3 |
| 60-69 | 4292 | 77.6 | 12.7 | 3389 | 75.5 | 12.4 | 2986 | 72.2 | 11.3 |
| 70-79 | 2800 | 78.9 | 12.8 | 2597 | 76.4 | 13.0 | 1353 | 72.5 | 11.4 |
| 80+ | 1215 | 79.4 | 13.0 | 994 | 77.2 | 13.5 | 584 | 73.2 | 11.5 |
| 20+ | 32,131 | 76.4 | 12.2 | 29,164 | 75.3 | 12.2 | 19,154 | 71.9 | 11.0 |
|  |  |  |  |  |  |  |  |  |  |
| 1. Men |  |  |  |  |  |  |  |  |  |
|  | HUNT1 | | | HUNT2 | | | HUNT3 | | |
|  |  | Heart rate | |  | Heart rate | |  | Heart rate | |
| Age | N | Beats/min | SD | n | Beats/min | SD | n | Beats/min | SD |
| 20-29 | 5836 | 72.4 | 11.9 | 3949 | 70.7 | 12.3 | 1516 | 69.4 | 11.2 |
| 30-39 | 7802 | 72.6 | 12.0 | 5274 | 70.3 | 11.7 | 2469 | 68.6 | 11.0 |
| 40-49 | 5616 | 73.2 | 12.2 | 6089 | 70.3 | 12.0 | 3702 | 68.1 | 10.9 |
| 50-59 | 4869 | 74.5 | 12.6 | 4524 | 71.2 | 12.4 | 3771 | 68.5 | 11.2 |
| 60-69 | 4789 | 74.9 | 12.9 | 3193 | 71.4 | 13.3 | 2638 | 68.0 | 11.7 |
| 70-79 | 3018 | 75.3 | 13.3 | 2518 | 71.9 | 13.5 | 1197 | 67.1 | 11.9 |
| 80+ | 1104 | 76.2 | 13.4 | 787 | 73.3 | 13.9 | 436 | 68.7 | 12.9 |
| 20+ | 33,034 | 73.7 | 12.4 | 26,334 | 71.0 | 12.4 | 15,730 | 68.3 | 11.3 |
